# Two-dimensional material-polymer hybrid membranes for enhanced mechanical stability and environmental remediation

**DOI:** 10.1186/s11671-026-04492-y

**Published:** 2026-03-06

**Authors:** Neetu Talreja, Divya Chauhan, Mangalaraja Ramalinga Viswanathan, Werayut Srituravanich, Mohammad Ashfaq

**Affiliations:** 1 Department of Science, Faculty of Science and Technology, Alliance University, Bengaluru, Karnataka 562106 India; 2 Department of Drinking Water and Sanitation, Ministry of Jal Shakti, 1208- A, Pandit CGO Complex, Lodhi Road, New Delhi, 110003 India; 3 Faculty of Engineering and Architecture, Universidad Arturo Prat, Avenida Arturo Prat 2120, 1110939 Iquique, Chile; 4 Department of Mechanical Engineering, Faculty of Engineering, Chulalongkorn University, Pathumwan, Bangkok, 10330 Thailand; 5 Department of Biotechnology, School of Sciences, Woxsen University, Hyderabad, Telangana 502345 India

**Keywords:** Membranes, Polymers, 2DMs, Water treatment, Mechanical stability, MXene

## Abstract

Two-dimensional materials (2DMs) have attracted substantial attention due to their exceptional thermal, tensile, chemical, and mechanical properties, making them promising candidates for designing next-generation polymeric membranes. Incorporating 2DMs with polymers not only improved selectivity and permeability but also considerably enhanced mechanical robustness, thereby improving long-term stability and durability, which are among the greatest challenges today. This review article provides new insights into advancements in 2DMs and 2DMs-based polymeric membranes, with particular emphasis on mechanical strength and their roles in environmental remediation. We also discussed how different 2DMs interact with polymers to address issues such as anti-fouling, stability, and durability in polymeric membranes. Additionally, we explore the challenges associated with polymeric membranes and how we can overcome them by using different 2DMs to develop a multifunctional 2DMs-polymer hybrid membrane that bridges mechanics with environmental remediation applications.

**Supplementary Information:**

The online version contains supplementary material available at 10.1186/s11671-026-04492-y.

## Introduction

Presently, two-dimensional materials (2DMs) such as graphene, graphene oxide (GO), graphitic carbon nitride (gC_3_N_4_), tungsten sulfide (WS_2_), hexagonal boron-nitride (h-BN), and MXene etc., have gained a substantial attention due to their excellent features such as exceptional temperature resistance ability, tensile strength, chemical resistivity, and mechanical strength etc., thereby effectively used for the several applications like environmental remediation, energy, sensor, agriculture, solar cell, and biomedical [1–8]. Additionally, the high surface area and excellent pore-size distribution of 2DMs make them an excellent candidate for environmental remediation applications. Numerous contaminants, including organic pollutants, dyes, heavy metal ions (HMIs), and pharmaceutical compounds, have increased significantly with industrialization and urbanization. These contaminants have adverse effects on humans, animals, and the ecosystem [9–12]. Therefore, there is a requirement to remove or degrade environmental contamination from environmental bodies.

Although 2DMs and their composites have been utilized for several processes, such as adsorption, oxidation/reduction, bioremediation, membrane separation technology, and advanced oxidation processes [13–15]. Among all of them, the adsorption and photocatalysis processes have attracted significant interest from researchers due to their rapidity and cost-effectiveness. However, these processes cannot achieve maximum adsorption/degradation of pollutants and sometimes produce toxic byproducts that remain in the solution, which again demonstrate toxic effects, and complete removal/degradation is another challenge [16–18]. In this context, membrane technology is essential for separating all types of pollutants, leaving no toxins in the solution.

Recently, a wide variety of membranes have been synthesized for water clean-up, as membrane-based technology offers easy separation based on the size of the pollutant. Most of the time, degradation efficiency is not a concern; material stability is. On the one hand, membrane technology offers several advantages for environmental remediation; on the other hand, it has several disadvantages, including membrane degradation, which is a major limiting factor [19–21]. In this context, improving the membrane’s mechanical properties is significant, and there is also an urgent need to identify methods that can significantly enhance its mechanical strength. Another concern with membrane technology is membrane fouling. Maintaining the mechanical strength of the polymeric membrane is a major challenge. In this context, a variety of 2DMs have been incorporated into the polymeric matrix to synthesize a membrane, thereby providing stability and high pollutant-degradation efficiency.

**Fig. 1:**
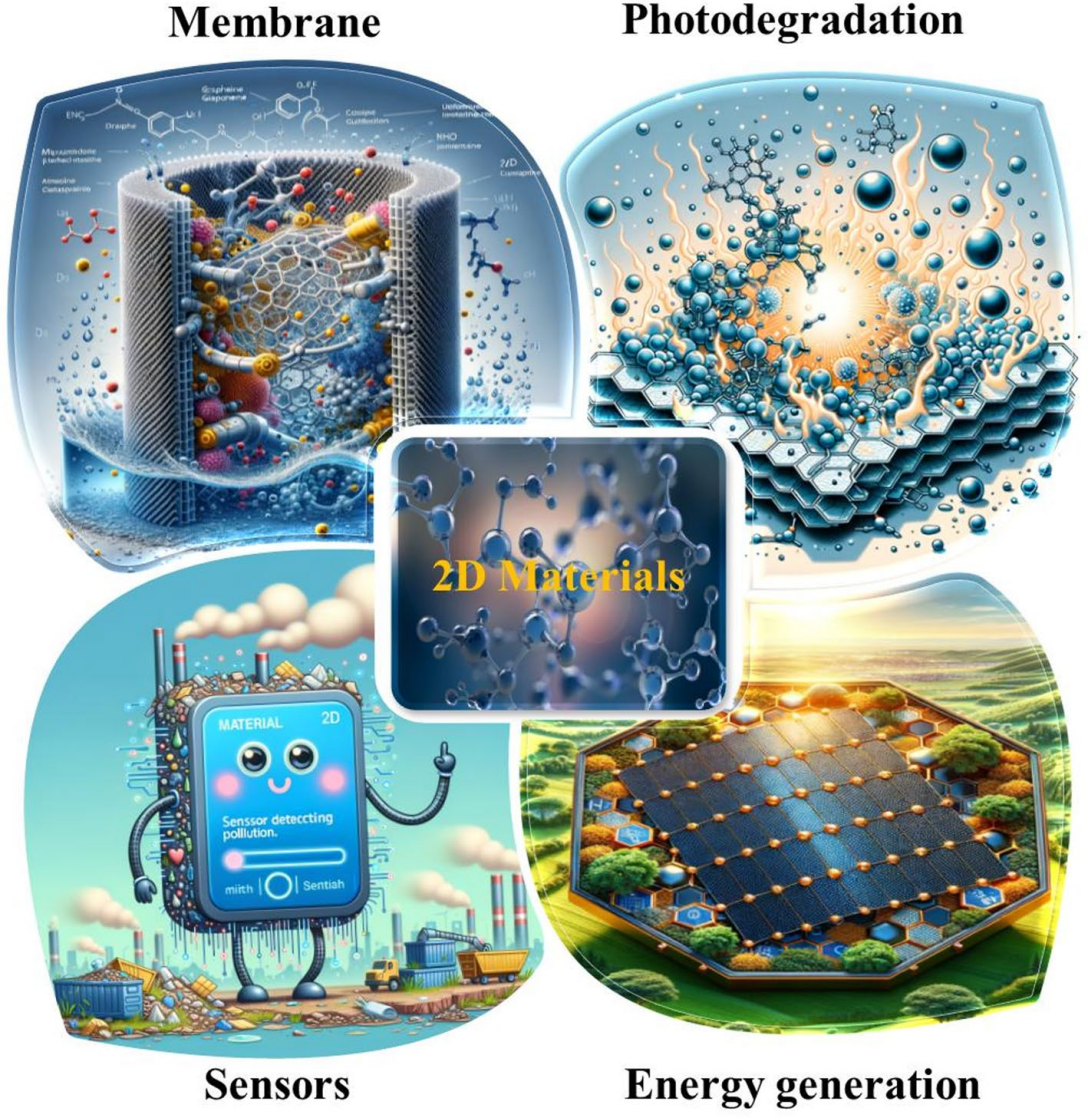
A schematic representation of the 2DMs in different areas of research, including membranes. Microsoft Copilot produced the artwork

Numerous 2DMs have been used in combination with polymers to degrade environmental pollutants. Some common examples of 2DMs for membrane synthesis include graphene, GO, rGO, transition metal dichalcogenides (TMDs, e.g., MoS_2_, MoSe_2_, WS_2_, and WSe_2_), h-BN, C_3_N_4_, MXene layers, and black phosphorus (BP) or phosphorene. 2DMs inherently exhibit a wide range of physico-chemical properties, from conducting graphene to semiconducting MoS_2_ to insulating h-BN. Moreover, the 2D crystal formations exhibit a unique combination of mechanical properties, with high in-plane stiffness and strength but very low flexural rigidity, thereby enabling easy degradation/removal of environmental pollutants [1, 11, 22–32]. Figure 1 shows a schematic representation of 2DMs across different research areas, including membranes. Here, we focus on the effects of 2DMs’ mechanical properties on the removal/degradation of environmental pollutants. We also discussed how 2DMs enhance the mechanical strength of polymeric membranes, thereby improving membrane stability and contaminant removal/degradation. Therefore, incorporating 2DMs into polymeric membranes might be a long-term solution for wastewater clean-up.

## Do mechanical properties affect the removal/degradation of pollutants?

The mechanical stability of a composite is useful for withstanding applied loads or pressures. Several terms, including ductility, hardness, impact resistance, fracture toughness, and tensile strength, can be used to describe a material’s mechanical properties. A few variables that affect the characteristics of the materials mainly are (1) grain boundaries (GBs), (2) surface functional groups, (3) point defects, and (4) dislocations. (1) The GBs are 1D/2D defects that play a significant role in determining the characteristics, especially mechanical, electrical, and optical [33–35]. Usually, upon mechanical strains (stretching and bending) in any material, including 2DMs, atomic structures change, leading to lattice vibrations, alterations in the electronic structure, changes in bandgap values, and changes in conductivity. Interestingly, monolayer graphene can withstand around 25% of strain without breaking [36–38]. Moreover, GBs in graphene and the interaction between interlayer mechanical characteristics, such as elasticity and fracture resistance, might be among the reasons for the weakening of fracture, thereby significantly affecting the mechanical strength of the materials/graphene [39, 40]. (2) The surface functional groups, graphene and their derivatives, including GO, have intrinsic characteristics and Young’s modulus of roughly around 76–293 MPa and 6–42 GPa, respectively. Their oxidized surface is the cause of the notable difference in the mechanical properties of graphene and GO. The presence of many oxygen groups on GO’s surface supports this considerable variation in mechanical properties. The difference between the mechanical strength of ordered GO and amorphous GO, and the key mechanical parameters (elastic modulus, E, and significant characteristics, sc) of the GOs generally decrease with increasing coverage of the functional groups [41–43]. 2DMs such as graphene, GO, rGO, TMDs, C_3_N_4_, and MXenes are indeed considered mechanically robust materials, since graphene has an intrinsic strength of 130 GPa and a Young’s modulus of 1.0 TPa; therefore, it is a potential candidate for various applications, including environmental remediation. (3) The point defects are lattice defects of zero dimensionality, as they do not possess a lattice structure in any dimension. Numerous 2DMs, including graphene, WS_2_, MoS_2_, hBN, phosphorene, and others, are known for having exceptional mechanical strength. The primary cause of the significant variation in their mechanical strengths is the presence of point defects, mostly in their crystals. As different forms of vacancies may be present in those point groups, like in case of MoS_2,_ there are various sorts of vacancies that can occur for example: mono-valency, bivalency, vacancy caused by a single Mo between three sulfurs, and vacancies caused by three di-sulfur pairs near Mo and Mo replacing S_2_ creates vacancy [44–47], and (4) dislocation, is one kind of defect that results from an atom moving away from its equilibrium position. A slight alteration in the configuration of these atoms results in a defect that alters the material’s mechanical characteristics. TMDs have concave three-dimensional (3D) polyhedra of polyelemental composition and generally exhibit dreidel-like shapes. Due to elemental heterogeneity, MoS_2_ has both Mo-rich and S-rich types, with a homo-elemental bond dislocation core, a typical dislocation structure in 2D materials composed of hexagonal rings. The dislocation site is unstable, however, as one has an M-rich core and the other an S-rich core, to preserve elemental balance. The insertion, substitution, or deletion of specific atoms in dislocations can yield derivative dislocation cores in MoS_2_. The formation energies of these derivative defects are related to the chemical equilibrium conditions. Dislocation can be confirmed by direct atomic-resolution imaging [48–50]. In general, the mechanical characteristics of 2DMs are essential, and incorporating 2DMs into membranes for water filtration can enhance membrane mechanical strength through mechanisms involving defects, grain boundaries, and other factors.

## 2DMs-based polymeric membranes for the removal of environmental contaminants

Usually, 2DMs change their mechanical properties, structure, and stability, and, with high mechanical strength and stability, maintain structural integrity during photocatalytic reactions. Because of this, 2DMs and their composites will remain effective and can also withstand additional degradation cycles, making them a sustainable material for photocatalytic applications. However, low mechanical strength leads to structural damage, reducing the degradation efficiency as a membrane and making it a poor candidate for photodegradation [51–53]. Moreover, the higher mechanical flexibility of 2DMs-based membrane polymer composites with surface roughness enables the higher adsorption capacity for various types of pollutants due to this roughness, the interaction between adsorbed pollutants and photogenerated reactive species is increased, such as electrons and holes, resulting in the higher photocatalytic degradation on the membrane surface [54–56]. Controlled manipulation of mechanical strain or defect density in 2DMs can change the charge carrier mobility, which ultimately improves the charge transport within 2DMs. The mechanical strain engineering and defect engineering strategies can modulate the electronic band structure, surface reactivity, and catalytic activity of 2DMs, which promotes the separation and migration of photogenerated electron-hole pairs, reducing the charge recombination and enhancing the generation of reactive oxygen species (ROS) responsible for enhancing the salt rejection properties of the membrane for solar light active membranes [57, 58]. Interestingly, the point-group symmetry of 2DMs can influence salt rejection during membrane separation under photo irradiation, as materials with higher symmetry may exhibit directional charge transport and enhanced light absorption in specific crystallographic directions, thereby improving photocatalytic performance [59, 60].

In summary, incorporating 2DMs into the polymer matrix is crucial for determining both mechanical strength and salt-rejection efficiency. In this context, advanced 2DM-based polymeric membranes with high mechanical strength and improved separation performance may be a good solution to global water issues [61–64]. Indeed, the mechanical properties of 2DMs-based membranes play a significant role in wastewater treatment. The incorporation of 2DMs into a polymeric matrix increases the membrane’s mechanical strength, as little deformation of the crystal structure is critical for maintaining structural stability and directly influencing the photodegradation efficiency of various pollutants.

### Graphene and its derivatives-based polymeric membranes

**Fig. 2:**
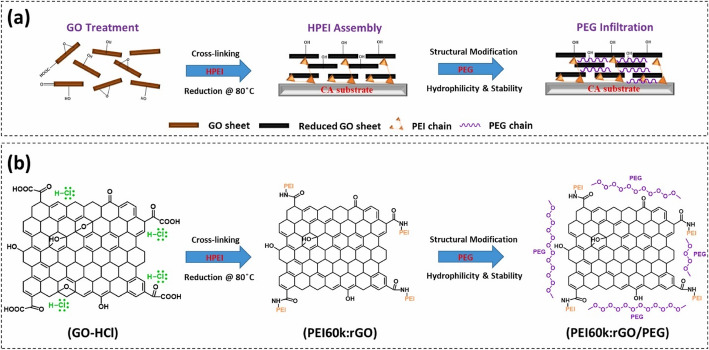
A schematic representation of the synthesis and mechanism of the PEG-GO-based membrane **a** synthesis process, and **b** mechanism. The image was reproduced with permission under the common creative license [68]

Graphene and its derivatives, such as GO, rGO, and functionalized graphene, are emerging materials that are transforming polymeric membrane technologies for water treatment. Indeed, the exceptional characteristics, such as the high surface area and tunable surface chemistry of graphene and its derivatives, significantly enhance adsorption capacity, ion rejection, mechanical stability, and anti-fouling behavior when incorporated into a polymeric matrix. Therefore, graphene and its derivatives are extensively used in polymeric membranes for environmental remediation [65–67]. Several studies have been conducted to date on the synthesis of graphene- and derivative-based polymeric membranes for environmental remediation applications. For instance, Edokali et al. developed a polyethyleneimine (PEI) and rGO-based membrane to enhance its stability and anti-fouling properties. Moreover, the mechanical stability of the membrane was improved by utilizing innovative electro-oxidative techniques. This process involved constructing PEI-crosslinked rGO layers on flat-sheet substrates functionalized with polyethylene glycol (PEG) and poly(3,4-ethylenedioxythiophene)-poly(styrene sulfonate) via electrophoretic deposition. Figure 2 shows the schematic representation of the synthesis and mechanism of the PEI-PEG-GO-based membrane. Cross-linking PEI with rGO enhances the membrane’s structural integrity. PEI provides multiple amine groups that can form strong covalent bonds with the oxygen-containing functional groups on GO, thereby yielding a more robust, stable membrane structure. Covalent cross-linking can reduce the number of point defects (such as vacancies and interstitials) in the GO structure. Fewer point defects mean the material has fewer weak spots that can initiate fractures, thereby enhancing its overall mechanical strength. Furthermore, the presence of strong covalent bonds at grain boundaries (interfaces between different crystalline regions within graphene sheets) can strengthen them. GBs are often points of weakness where fractures can propagate by reinforcing these boundaries with covalent bonds, the material becomes more resistant to mechanical deformation and cracking. Additionally, the introduction of a cross-linked network can act as a barrier to dislocation movement. Dislocations are defects in the crystal structure that can move under stress, leading to plastic deformation. The covalent bonds and cross-linked network can pin these dislocations, making it more difficult for them to move. It results in a higher resistance to plastic deformation and, consequently, an increase in mechanical strength. Also, the cross-linked network adds rigidity to the GO structure, thereby reducing its elastic deformation. This rigidity further enhances the membrane’s mechanical stability, ensuring it maintains its shape and integrity under stress [68].

Noreen et al. synthesized three different polymer-based GO nanocomposites (GO/polyaniline (PAN), GO/polypyrrole (PPy), and GO/polystyrene (PS)) for the treatment of dyes in industrial wastewater. The incorporation of GO into the polymer matrix increases the adsorption efficiency of the nanocomposites due to its high surface area and functional groups that can interact with dye molecules. Additionally, the 2D structure of GO facilitates better dispersion within the polymer matrix, resulting in more uniform composite materials. Overall, GO’s excellent mechanical properties contribute to the overall strength and durability of the composite materials [69]. Another study by Elzubair et al. dispersed GO in a PTFE-based membrane and then used it for desalination. The incorporation of GO into the polymer matrix enhances the membrane’s overall mechanical strength. The functional groups in GO, such as carboxyl, hydroxyl, and epoxy groups, can form strong interfacial interactions with the polymer matrix, thereby enhancing the mechanical integrity of the composite. Also, cross-linked networks can further enhance the mechanical strength. Cross-linking creates a more interconnected, rigid structure, thereby improving the membrane’s durability [70].

Goyat et al. synthesized polyethersulfone (PES) membrane adorned with GO-TiO_2_ nanocomposites. The incorporation of GO not only increases mechanical strength but also increases antibiotic degradation efficiency from 66.52% to 89.81%. Further, the addition of polyvinylpyrrolidone (PVP) provides high corrosion resistance, flexibility, and durability [71]. Another study by Akin et al. reported the synthesis of a cyclodextrin-modified GO-incorporated polymer membrane. In the synthesized composite, the arsenate rejection rate was 87.6% higher than that of other salts [72]. Sun et al. developed a pH-responsive membrane by incorporating GO into the PMMA matrix. They applied it to reject negatively charged dyes (MO and EB) under acidic conditions. Carboxylic groups on PMMA and GO interact, altering the interlayer spacing of GO sheets and improving the membrane’s performance and mechanical stability [73]. These studies suggested that graphene and its derivatives exhibit exceptional mechanical properties and serve as fillers that contribute to the overall strength, durability, separation efficiency, and anti-fouling properties of the polymeric membrane. Additionally, despite tremendous success, cost-effective synthesis, a process for large-scale production, defect-free graphene derivatives, uniform dispersion within the polymeric matrix, longer-term stability, and environmental safety remain concerns.

### TMDs-based polymeric membranes

**Fig. 3:**
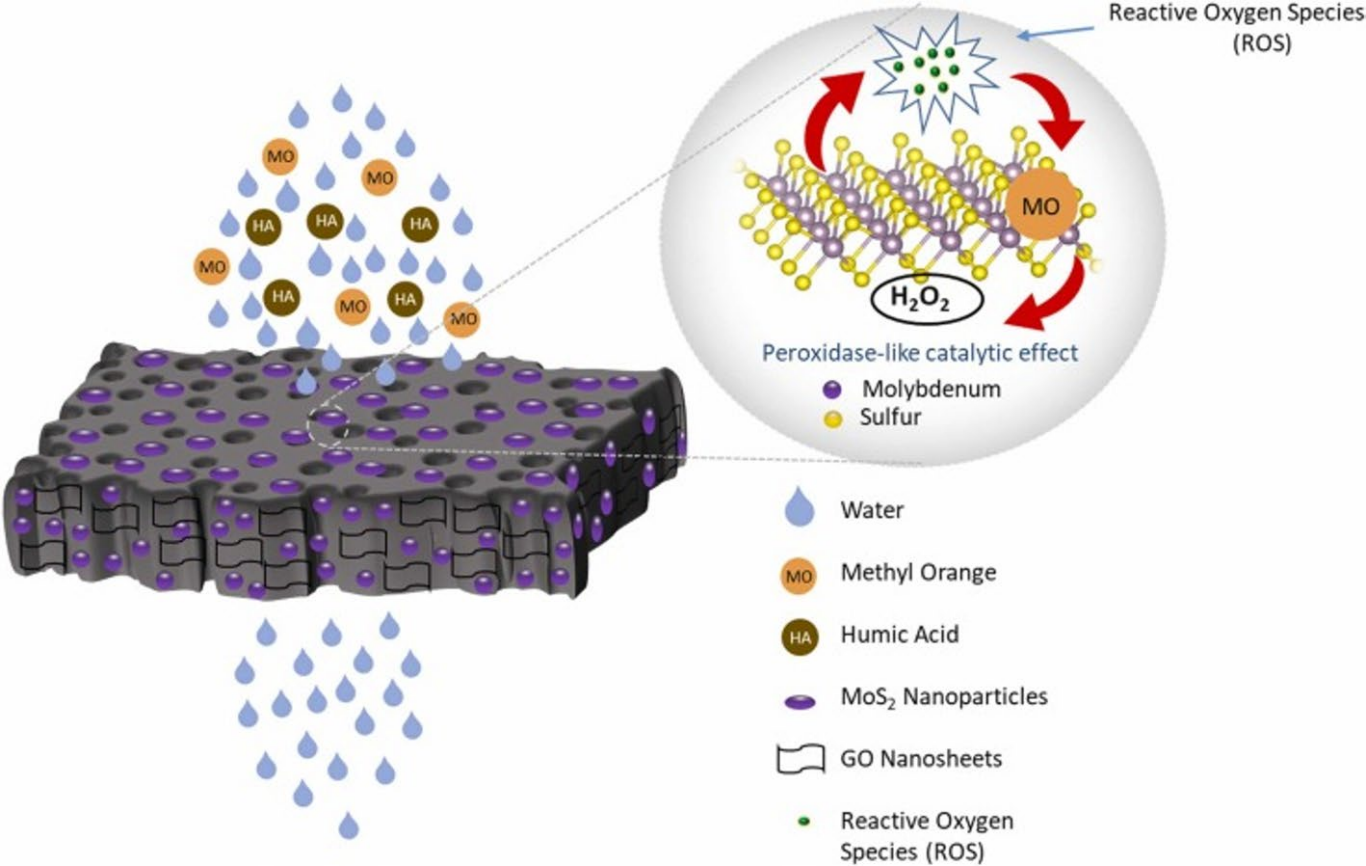
A schematic illustration of a chitosan/GO/MoS_2_ membrane for water treatment. The image was reproduced with permission under the common creative license [75]

TMDs like MoS_2_, MoSe_2_, WS_2_, and WSe_2_ have emerged as promising candidates for environmental remediation applications due to their exceptional physicochemical properties and tunable surface chemistry. The incorporation of TMDs into the polymeric matrix significantly enhances membrane performance by creating micro- and nanochannels for rapid mass transfer and improving selectivity towards analytes. Furthermore, TMD-based polymeric membranes exhibit exceptional mechanical strength, thermal stability, and anti-fouling properties, which are crucial for long-term operation. Numerous studies have been conducted to date focusing on the synthesis of TMD-based membranes for the removal of environmental contaminants. For instance, Yang et al. mentioned the mechanical properties of 2DMs and their impact on photocatalysis. According to the study, TMDs, like MoS_2_ and WS_2_, are typically mechanically robust materials with strong interatomic bonds. The strong bonding provides structural stability, ensuring that all TMD-based photocatalysts maintain their integrity under harsh environmental conditions, including exposure to light, moisture, and temperature fluctuations, thereby enabling long-term performance in photocatalytic reactions. The authors also reported that applying controlled mechanical strain or deformation to TMDs can modulate bandgap energy, charge-carrier mobility, and surface reactivity, thereby improving photocatalytic efficiency for pollutant degradation. Furthermore, the kinetics of charge carrier generation, separation, and transport also depend on the mechanical flexibility of TMDs, which can facilitate the migration of photogenerated charge carriers to the material surface, where they participate in redox reactions with adsorbed pollutant molecules, leading to higher degradation. Moreover, the formation and distribution of defects in TMDs can serve as active sites for the photocatalytic reactions. The defect engineering strategies, such as introducing vacancies, dopants, or grain boundaries, can enhance the photocatalytic activity of TMDs by promoting the generation of ROS and facilitating the activation of molecular oxygen during pollutant degradation [74]. Momani et al. synthesized a chitosan/MoS_2_/GO composite membrane and tested its degradation of organic contaminants [75]. Figure 3 shows the schematic illustration of a chitosan/GO/MoS_2_ membrane for water treatment. The 2DMs, such as GO and MoS_2_, within the polymeric membrane significantly improved stability and degradation ability.

Zhao et al. synthesized a P-PVDF/MoS_2_ membrane and used it for the removal of Hg ions from wastewater. The composite was synthesized by blending MoS_2_ into a PVDF matrix. The PVDF provides porous skeleton support to the MoS_2_, which ultimately increases the mechanical strength of the developed membrane [76]. Oskoui et al. synthesized a MoS2/O-MWCNTs nanocomposite via a hydrothermal process, then blended it with polyethersulfone to develop a membrane, and applied it for the removal of dyes, antibiotics, and proteins such as bovine serum albumin, achieving a high removal efficiency of 99%. According to the study, acid-functionalized MWCNTs contain several functional groups, such as -OH and -COOH, which could facilitate the uniform dispersion of MoS_2_ in the polymer matrix. This blend alters the membrane’s properties, including mechanical strength, permeability, and low fouling [77]. Another study by Rodrigo Schneider et al. immobilized MoS_2_ nanoflakes onto poly (lactic acid) (PLA). By the solution blow technique and then coated with Zein and spray-dried with MoS_2_ nanoflakes. The synthesized membrane was then used for the removal of the methylene blue dye, with a high adsorption capacity of 112 mg g^− 1^ [78]. The incorporation of MoS_2_ into a polymer matrix can improve the membrane’s ability to maintain its shape and functionality under mechanical stress. Additionally, integration of MoS_2_ nanoflakes into the PLA matrix can affect mechanical stability. Uniform dispersion and alignment of the nanoflakes within the membrane can enhance mechanical properties by preventing stress concentration in specific areas, thereby reducing the likelihood of failure [78]. Yang et al. added MoS_2_ nanosheets to a polyamide matrix to prepare a thin-film polymer-based membrane. Due to cross-linking between MoS_2_ and polyamide (PA), a robust network structure forms, further increasing the mechanical stability of the membrane [79].

Keshebo et al. developed a polyether sulfone membrane doped with WS_2_ nanosheets to increase solute rejection rates for both bovine serum albumin (BSA) and humic acid from 89 to 98%. The addition of WS_2_ to the polymer matrix increases the membrane’s durability, making it more resistant to mechanical wear and tear. The presence of WS_2_ nanosheets reduces the membrane’s susceptibility to physical stress-induced damage. Also, the membrane’s toughness, a measure of its ability to absorb energy before failure, is improved. This is due to the combined effects of enhanced tensile strength and elastic modulus, as well as the energy dissipation mechanisms provided by the addition of WS_2_ nanosheets [80]. Aqaei et al. synthesized a polyvinyl chloride (PVC) membrane incorporating WS_2_ nanosheets to improve its hydrophilicity, porosity, permeability, and fouling resistance. The synthesized membrane showed improved performance for various salt rejections [81]. These studies suggested that TMDs, such as graphene and its derivatives, exhibit exceptional properties and exceptionally tunable surface chemistry. Moreover, incorporating TMDs into polymeric membranes by forming interfacial interactions and restricting polymer chain mobility subsequently enhances tensile strength and durability. Indeed, improved mechanical strength enables polymeric membranes to withstand high pressure and harsh conditions, enabling efficient, long-lasting performance in environmental remediation.

### MXene-based polymeric membranes

**Fig. 4:**
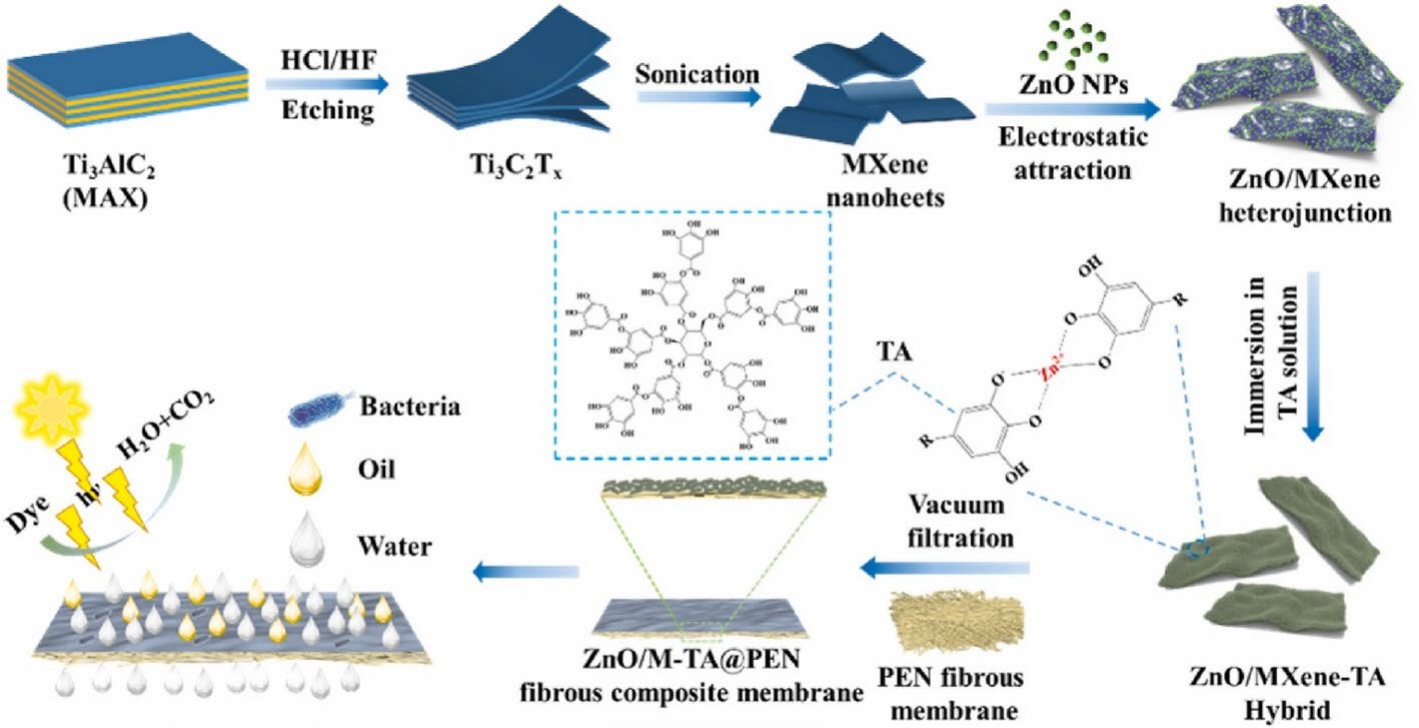
A schematic illustration of the synthesis of the MXene/ZnO-based membrane for water treatment. The image was reproduced with the permission [92]

Like other 2DMs, MXene has been used to improve mechanical strength and environmental remediation due to its high aspect ratio. The incorporation of MXene creates a large interfacial area within the polymer matrix, thereby improving load transfer between the MXene sheets and the polymer [82, 83]. Such interaction enhances the mechanical strength and toughness of the composite membrane. Additionally, MXenes, which contain several functional groups like -OH, -O, and -F, can form strong interfacial bonds with the polymer matrix. This improved interfacial adhesion helps in efficiently transferring stress between the polymer and the filler, resulting in better mechanical performance [84–87]. Several studies report the incorporation of MXene into polymer matrices to enhance mechanical performance. For instance, Xiaotong Mu et al. reported the synthesis of an MXene/Ag_3_PO_4_/PVDF-modified membrane, in which the designed junction promotes electron transfer and charge separation. Moreover, the uniform dispersion of MXene within the PVDF matrix ensures that mechanical load is more evenly distributed throughout the composite. This uniform distribution of stress helps in preventing localized failure points, thereby enhancing the overall mechanical properties of the membrane [88]. Another study by Zhang et al. synthesized MXene/poly-melamine-formaldehyde composite membranes and applied them for the removal of heavy metals. In this study, MXenes act as reinforcing agents within the polymer matrix. The presence of MXenes restricts the mobility of the polymer chains, thereby increasing the rigidity and strength of the composite. This ultimately increases the mechanical strength of the composite membrane [89]. Nan Li et al. synthesized polyaniline and MXene composites, which were then mixed into a polyethersulfone matrix to form a strong 3D network with high mechanical strength and high dye degradation [90]. Imsong et al. synthesized a super-leophobic MXene-PAN membrane for oil-water separation, which can also adsorb organic pollutants in an oily environment. The membrane is also stable in a highly corrosive environment. The high stability is due to the incorporation of MXene, which increases the material’s strength [91]. Sun et al. synthesized tannic acid modified MXene/ZnO heterojunction and self-assembled onto the poly (arylene ether nitrile) to form a membrane and used for oil water separation [92]. Figure 4 shows the schematic illustration of the synthesis of the MXene/ZnO-based membrane for water treatment. Interestingly, the incorporation of MXene into the membrane not only increases mechanical strength but also degradation resistance.

Overall, MXene enhances the mechanical properties of polymer-based membranes through its high aspect ratio, inherent strength, improved interfacial adhesion, uniform dispersion, and reinforcement effect. These factors collectively contribute to a more mechanically robust composite membrane, suitable for applications that require both mechanical strength and functional performance, such as interfacial evaporation and photodegradation.

### C_3_N_4_ and h-BN-based polymeric membranes

**Fig. 5:**
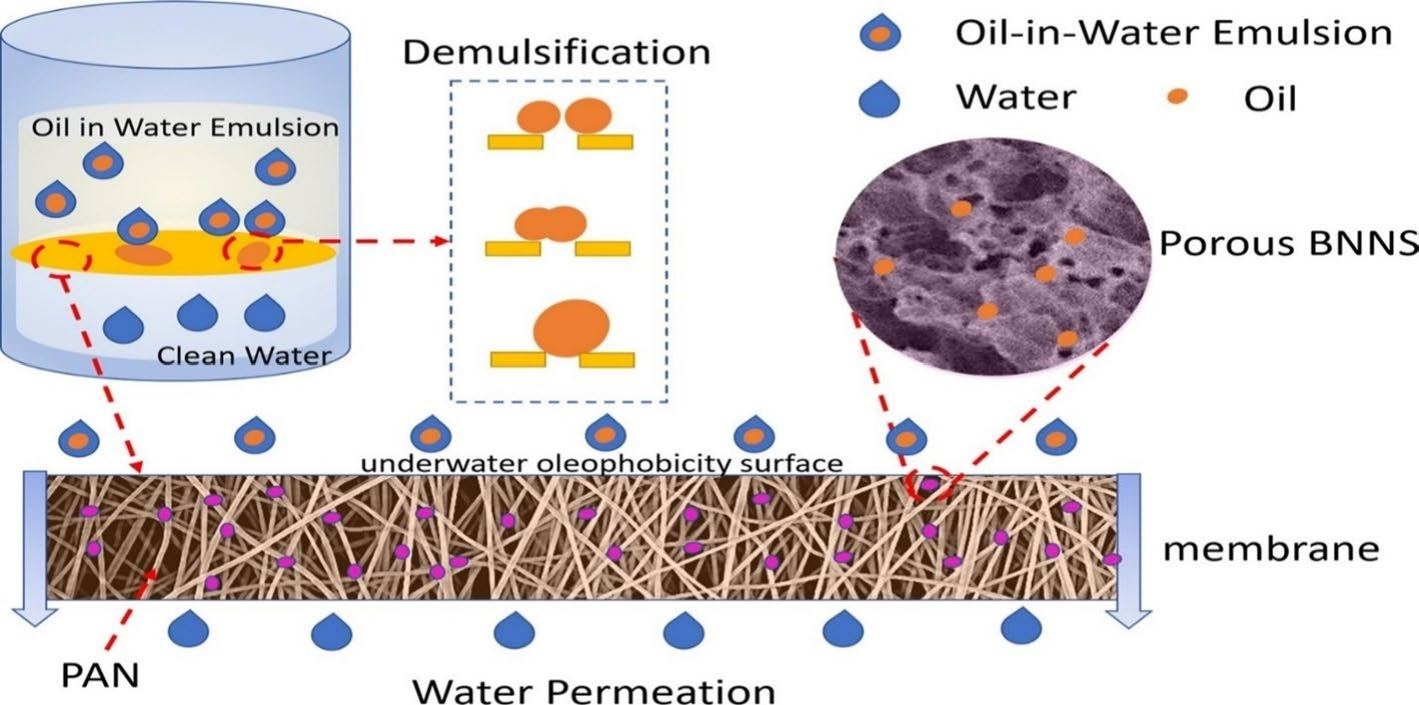
A schematic representation of the separation mechanism from an oil-in-water emulsion. The image was reproduced with permission [94]

C_3_N_4_ and BN-based 2DMs have also been incorporated into a polymer matrix to improve the strength and performance. Several studies report composites of C_3_N_4_ and BN with polymers, such as those by Karabo C. Mashiloane et al., who synthesized h-BN incorporated with PVDF and applied it for oil-water separation. The uniform dispersion of h-BN within the PVDF matrix prevents the formation of stress concentration points. This uniform distribution of h-BN nanosheets ensures that the mechanical stress is evenly distributed across the membrane. The presence of h-BN can hinder the propagation of cracks within the PVDF matrix. The h-BN nanosheets create a tortuous path for cracks, increasing the energy required for crack propagation and thereby enhancing the membrane’s fracture toughness. The addition of h-BN can influence the crystallization behavior of PVDF. The h-BN nanosheets can act as nucleation sites for the polymer, increasing crystallinity and forming more stable crystalline phases, thereby improving mechanical properties such as tensile strength and modulus [93]. Li He et al. developed an h-BN/PAN composite membrane and applied it for oil-water separation. The synthesized membrane exhibited outstanding mechanical properties due to the incorporation of h-BN, which altered its morphology, creating a more compact, interconnected structure. This improved morphology can enhance the mechanical integrity of the membrane [94]. Figure 5 shows the schematic representation of the separation mechanism from an oil-in-water emulsion. The incorporation of h-BN significantly improved the porous texture of the materials, thereby achieving a high separation efficiency. These studies suggest that CN and h-BN significantly improved the mechanical stability and performance of the polymeric membranes. However, there is still a need to enhance polymeric membranes that exhibit exceptional mechanical stability and high removal of environmental pollutants.

Table 1 summarizes the different 2DMs within the polymeric membranes. The researchers are continuously increasing the mechanical stability and performance of the polymeric membranes. Indeed, incorporating 2DMs into polymeric membranes provides new insights into environmental remediation. Another strategy is to use atomic doping (boron (B) and fluorine (F)) in 2DMs, which might be a long-term solution that significantly enhances structural and functional properties. Interestingly, atomic doping (e.g., B and F) substantially modifies the electronic structure and strengthens the covalent network, thereby conferring exceptional mechanical stability and resistance to structural degradation. Additionally, atomic doping substantially increases surface area, active sites, and charge-transfer efficiency, thereby improving adsorption, photocatalytic activity, and the degradation of environmental pollutants. For instance, Yuan-Yuan Li utilizes doping strategies to strengthen surface defects and ultimately enhance photocatalytic performance. The doped B and F in the skeleton of C_3_N_4,_ due to the incorporation of heteroatoms, introduce lattice distortions or defects, such as vacancies, dislocations, or grain boundaries, which can ultimately provide strength to the material, hinder dislocation movement, and increase resistance to deformation. These defects act as barriers to dislocation motion, thereby enhancing the material’s mechanical strength. The presence of grain boundaries in fine-grained materials can promote the separation and migration of photogenerated charge carriers. GBs act as sinks for charge carriers, preventing their recombination and extending their lifetime. This prolonged lifetime of charge carriers enhances the efficiency of photocatalytic reactions by allowing them to participate in redox processes for longer. Fine-grained materials often exhibit improved mass transport properties due to their high GB density. The GBs can serve as pathways for the diffusion of reactant molecules and the transport of photogenerated species to and from the material surface. Enhanced mass transport facilitates the supply of reactants and the removal of reaction products, thereby improving photocatalytic performance. The GBs in fine-grained materials can introduce additional surface defect sites, such as step edges and kink sites, which are highly reactive for catalytic reactions. These defect sites provide active sites for the adsorption and activation of reactant molecules, promoting the initiation of photocatalytic reactions and accelerating reaction kinetics. The high density of GBs in fine-grained materials acts as a barrier to charge-carrier diffusion, limiting their mobility and reducing the likelihood of recombination. This suppression of charge-carrier recombination enhances the quantum efficiency of photocatalytic reactions, thereby improving photocatalytic performance [95]. In this context, incorporating 2DMs into a polymer matrix provides the material with high strength, which further supports membrane stability and ultimately its performance.

**Table 1:** summarizes the different 2DMs within the polymeric membranes

S. No.	Membrane	2DMs	Remarks	Ref
1.	PEG-GO	rGO	Incorporation of rGO enhances the membrane’s structural integrity	[68]
2.	GO-PTFE	GO	GO significantly enhances the membrane’s mechanical strength	[70]
3.	GO-TiO_2_-PES	GO	The incorporation of GO not only increases mechanical strength but also increases antibiotic degradation efficiency	[71]
4.	GO-cyclodextrin	GO	The incorporation of GO into the membranes significantly improved mechanical strength, and the arsenate rejection rate was 87.6%	[72]
5.	PMMA-GO	GO	Carboxylic groups alter the interlayer spacing of GO sheets, improving the membrane’s performance and mechanical stability	[73]
6.	Chitosan/MoS_2_/GO	MoS_2_/GO	The incorporation of MoS_2_/GO into a membrane significantly improved mechanical strength and degradation resistance	[75]
7.	PVDF- MoS_2_	MoS_2_	PVDF provides porous skeleton support to the MoS_2_, which ultimately increases the mechanical strength	[76]
8.	F-MWCNTs-MoS_2_	MoS_2_	Surface functional groups -OH and -COOH facilitate the uniform dispersion of MoS2 in the polymer matrix, improving the membrane’s properties, including mechanical strength, permeability, and low fouling	[77]
9.	MoS_2_-PLA	MoS_2_	MoS_2_ nanoflakes in the PLA matrix can affect mechanical stability	[78]
10.	polyether sulfone membrane-WS_2_	WS_2_	The incorporation of WS_2_ within the polymer matrix increases the membrane’s durability, making it more resistant to mechanical wear and tear	[80]
11.	PVC-WS_2_	WS_2_	The incorporation of WS_2_ significantly improves its hydrophilicity, porosity, permeability, and fouling resistance	[81]
12.	MXene/Ag_3_PO_4_/PVDF	MXene	The incorporation of MXene within the membrane significantly improved the mechanical properties	[88]
13.	Super-leophobic MXene-PAN	MXene	The incorporation of MXene significantly increased the membrane’s mechanical stability	[91]
14.	PVDF-h-BN	h-BN	The incorporation of h-BN increases crystallinity and forms more stable crystalline phases, thereby improving mechanical properties such as tensile strength and modulus	[93]

## Challenges associated with 2DMs-polymeric membrane

Indeed, membrane technology struggles with fouling, a significant disadvantage of the membrane industry. To overcome this issue, several anti-fouling agents or nanomaterials, especially 2DMs, need to be incorporated into the polymer matrix, thereby significantly increasing fouling resistance. Further studies should be conducted to prevent microbial colonization on the membrane surface [96–98]. For this, metallic nanomaterials such as Cu, Zn, Ag, and Au can be incorporated into 2DMs, followed by polymeric membranes [99]. Moreover, when incorporating metallic nanomaterials into 2DMs, one of the most critical parameters to consider is that the interlayer spacing of 2DM nanosheets should not be too large; controlling this spacing is again challenging when incorporating 2DMs into polymer membranes [6, 100]. Moreover, the incorporation of 2DMs significantly increased the membranes’ porosity, which can be tuned to suit the pollutants.

Indeed, mechanical properties affect the removal/degradation of pollutants, particularly through polymers and their fillers, such as 2DMs. The study suggests that 2DMs-based membrane performance is not only governed by intrinsic chemical selectivity but also by mechanical stability under operating conditions [101]. The high permeability and mechanical stability of 2DMs, such as graphene, are remarkable, yet they suffer from pressure-induced compaction and interlayer collapse. In contrast, MXenes and other TMDs significantly enhance tunable surface chemistry and wettability. However, interlayer swelling, oxidation, and mechanical softness (phase-dependent) are some of the most significant challenges [102–105]. 2DMs like h-BN and pristine graphene possess high stiffness but require structural reinforcement at the membrane level, whereas g-C_3_N_4_ is mechanically brittle, thereby offering high-performance photo-responsive activity [4, 106–108]. Together, these insights suggested that future membrane design must move beyond permeability and selectivity towards integrated mechanical transport frameworks, where resistance to compaction shear and fatigue is clearly linked to sustained separation performance and the scalability of 2DM-based membranes.

## Conclusion

2DMs incorporated into polymers to form polymeric membranes is one of the most demanding areas of research today. Numerous 2DMs have been incorporated into the polymer membrane to enhance its strength and mechanical properties. The 2DMs have a high aspect ratio, and several functional groups on their surfaces enhance the membrane’s hydrophilicity, thereby improving its salt rejection properties. The incorporation of 2DMs into the polymer also increases the fouling resistance, thereby enhancing the life of the polymeric membrane. Also, the nanopores within the layers shorten the ion transfer distance, increasing their effectiveness for separation. In this context, 2DMs-based polymeric membranes are very effective for wastewater separation, providing mechanical strength through strong covalent bonding, high aspect ratios, and uniform dispersion. Therefore, incorporating 2DMs into a polymer matrix is a good strategy to improve performance and strength.

## Supplementary Information

Below is the link to the electronic supplementary material.

Supplementary Material 1.

Supplementary Material 2.

## Data Availability

No datasets were generated or analysed during the current study.
